# Phenolic Acids as Antidepressant Agents

**DOI:** 10.3390/nu14204309

**Published:** 2022-10-14

**Authors:** Maria Lúcia da Silva Cordeiro, Verônica Giuliani de Queiroz Aquino Martins, Ariana Pereira da Silva, Hugo Alexandre Oliveira Rocha, Vanessa de Paula Soares Rachetti, Katia Castanho Scortecci

**Affiliations:** 1 Laboratório de Transformação de Plantas e Análise em Microscopia (LTPAM), Departamento de Biologia Celular e Genética, Universidade Federal do Rio Grande do Norte (UFRN), Natal 59072-900, Brazil; 2 Programa de Pós-Graduação em Bioquímica e Biologia Molecular, Centro de Biociências, Universidade Federal do Rio Grande do Norte (UFRN), Natal 59072-900, Brazil; 3 Laboratório de Biotecnologia de Polímeros Naturais (BIOPOL), Departamento de Bioquímica, Universidade Federal do Rio Grande do Norte (UFRN), Natal 59072-900, Brazil; 4 Laboratório de Psicofarmacologia, Departamento de Biofísica e Farmacologia, Centro de Biociências, Universidade Federal do Rio Grande do Norte (UFRN), Natal 59072-900, Brazil

**Keywords:** phenolic compounds, medicinal plants, depression, behaviour, antioxidants, antiinflammatory

## Abstract

Depression is a psychiatric disorder affecting the lives of patients and their families worldwide. It is an important pathophysiology; however, the molecular pathways involved are not well understood. Pharmacological treatment may promote side effects or be ineffective. Consequently, efforts have been made to understand the molecular pathways in depressive patients and prevent their symptoms. In this context, animal models have suggested phytochemicals from medicinal plants, especially phenolic acids, as alternative treatments. These bioactive molecules are known for their antioxidant and antiinflammatory activities. They occur in some fruits, vegetables, and herbal plants. This review focused on phenolic acids and extracts from medicinal plants and their effects on depressive symptoms, as well as the molecular interactions and pathways implicated in these effects. Results from preclinical trials indicate the potential of phenolic acids to reduce depressive-like behaviour by regulating factors associated with oxidative stress, neuroinflammation, autophagy, and deregulation of the hypothalamic–pituitary–adrenal axis, stimulating monoaminergic neurotransmission and neurogenesis, and modulating intestinal microbiota.

## 1. Introduction

Depression is a mental disorder that affects approximately 3 to 5% of the population worldwide. This psychiatric disorder is the primary contributor to deaths by suicide, which total approximately 800,000 annually [1,2]. This disorder encompasses several emotional symptoms, such as sadness and persistent anhedonia, reduced interest of the person in their environment, psychomotor changes, such as fatigue, changes in sleep and body weight, and other neurocognitive effects, and physical symptoms [3,4].

**Figure 1:**
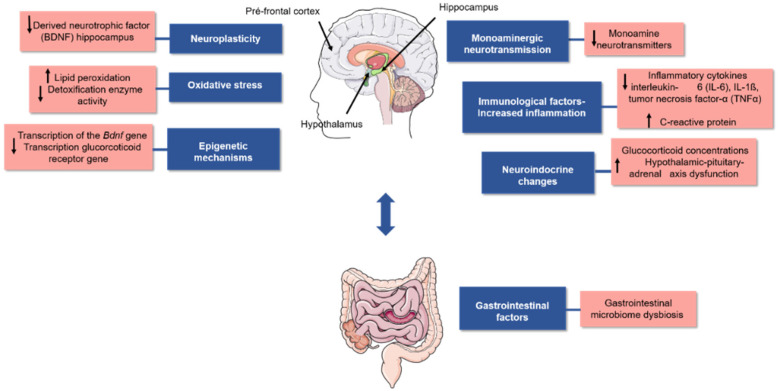
Factors associated with molecular pathophysiology of depression. Illustration of the brain regions involved in dysfunction in the pathophysiology of depression (prefrontal cortex, hippocampus, and hypothalamus). The regions are shown by black arrows and bidirectional communication between the gut (represented by a black arrow) and the human brain. This communication reinforces the importance of the gut microbiota in this neurological disorder. Because of its multifactorial nature verified in several studies, a summary of biological processes (highlighted in blue) and molecular factors (highlighted in red) associated with this disorder is presented in this image. Figure was created using images from Server Medical Art (https://smart.servier.com) accessed on 16 August 2022, licensed under a Creative Commons Attribution 3.0.

Although the reasons for this condition are not completely clear, several studies have proposed that it is associated with biological factors, such as changes in the levels of noradrenergic, serotoninergic, and dopaminergic neurotransmitters. These changes may be caused by the reuptake or degradation of monoamines in the synaptic cleft, reduction in neurogenesis, inflammation, oxidative stress, increased glucocorticoid concentration (e.g., cortisol), changes in gut microbial abundance and diversity, and epigenetic modifications in DNA. All these biological factors could correlate with the depression pathophysiology as represented in Figure 1 [5,6,7,8,9,10].

**Figure 2:**
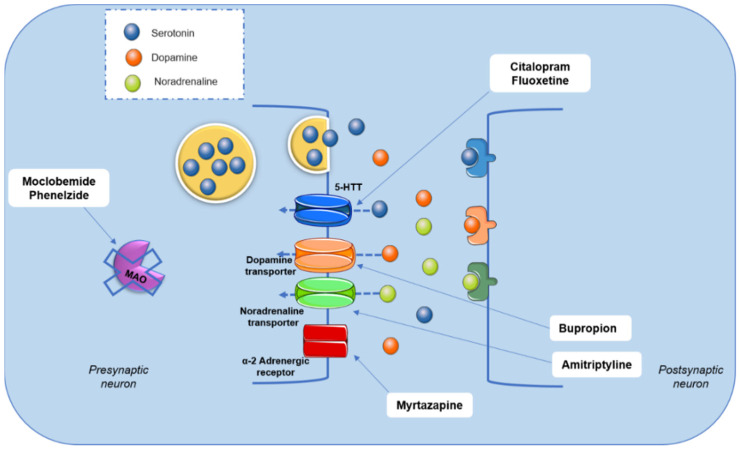
Mode of action of monoamine-modulating antidepressants. Selective serotonin reuptake inhibitors, such as fluoxetine, bind to the serotonin transporter (5-HTT) and block the reuptake of this neurotransmitter, whereas antidepressants, such as bupropion, belong to the group of noradrenaline and dopamine reuptake inhibitors. Tricyclic antidepressants, such as amitriptyline, prevent the reuptake of serotonin, dopamine, and noradrenaline in a non-specific manner by binding and blocking their transporters. The α2 adrenergic receptor antagonists, such as mirtazapine, bind to these receptors, increasing noradrenaline levels. Monoamine oxidase (MAO) enzyme inhibitors, such as phenelzine, bind to isoforms of the enzyme, inhibiting its binding to monoamines, thereby blocking its enzymatic catalysis. Figure was created using images from server Medical Art (https://smart.servier.com) accessed on 16 August 2022, licensed under a Creative Commons Attribution 3.0.

Although the mechanism of depressive disorder is not completely understood, pharmacological treatment primarily consists of drugs that modulate brain monoamine metabolism (Figure 2). Drugs, such as moclobemide and phenelzine, act through monoamine oxidase (MAO-A and MAO-B) inhibition. This pathway is associated with monoamines’ degradation. Tricyclic antidepressants, such as amitriptyline and nortriptyline, promote a non-selective inhibition of the reuptake of monoamines, mainly serotonin and noradrenaline. Selective serotonin reuptake inhibitors, such as fluoxetine and citalopram, noradrenaline and dopamine reuptake inhibitors (bupropion and reboxetine), and α2 adrenergic receptor antagonists, such as mirtazapine, are other options in the pharmacotherapy of depressive disorders [4,8,11,12,13] (Figure 2).

Depression is a multifactorial condition, and pharmacological treatment has limitations, such as the latency for the therapeutic effect (at least 3–4 weeks) and refractoriness to drugs [14,15,16]. It has been observed that some patients become resistant to these drugs, and a combination of drugs or a higher dosage is often necessary to obtain treatment results. Moreover, side effects, such as sexual dysfunction, weight gain, sleep disorders, dizziness, nausea, and lethargy lead to treatment dropout [4,17]. Thus, in the search for alternative strategies to increase treatment efficiency, different molecular pathways associated with the development of depression have been examined [12,18].

Among treatment strategies, medicinal plants are a biological resource of substantial relevance [19]. Plants produce several phytochemicals, known as secondary metabolites, as an adaptive response to environmental conditions. Additionally, different bioactive molecules have been characterised by their applications in human health. The prospection and characterisation of old/new bioactive molecules is an important research field based on its application and the production of new drugs in the pharmaceutical, nutritional, and cosmetic industries [20,21,22]. Phytochemicals may be divided into three major groups: alkaloids, terpenes, and phenolic compounds [21]. Some bioactive molecules that are of importance to global health include atropine (indicated to combat heart arrhythmias and ulcers), codeine and morphine (used as analgesics), camptothecin (antineoplastic activity), ergotamine (migraine treatment), and quinine (malaria treatment) [20,23,24].

Several studies have shown that the bioactive molecules from medicinal plants and isolated compounds have potentially important psychopharmacological and neuroprotective effects and antidepressant potential by acting in different pathways [19,25,26,27,28]. While considering the three major groups of phytochemicals, in this review, the focus is on phenolic acids, one of the main classes of phenolic compounds. Because of the chemical structure of phenolic acid, this bioactive molecule also has antioxidant activity. This molecule is widely present in fruits, vegetables, and medicinal plants [29,30].

Oxidative and nitrosative stress are considered a redox imbalance in cells and tissues promoted by an increase in reactive oxygen species (ROS) or reactive nitrogen species (RNS) and/or by reducing endogenous antioxidant defence. This characteristic is associated with the development of different chronic diseases [31]. Moreover, oxidative and nitrosative stress play important roles in developing psychiatric disorders, such as depression (Figure 1). The antioxidant nature of phenolic acids makes them important bioactive molecules with the potential to prevent neurological diseases and as an important strategy for their treatment [12,27].

Phenolic acids are an important part of our diet because these bioactive molecules are present in different natural foods, herbs, and spices (e.g., thyme), fruits, nuts, tubers, vegetables, cereal grains, and beverages, such as different types of tea and coffee [32]. Studies have shown that the consumption of vegetables and fruits could be associated with antioxidant protection and the prevention of depression in most people [7,33,34]. The relationship between polyphenol compounds primarily from diet and the development of depression was verified by [35]. In their work, the ingestion of phenolic acids such as caffeic, ferulic, and vanillic acid, flavanones, and anthocyanins had a significant protective effect in preventing the development of depressive symptoms. Therefore, this review focuses on the results of preclinical studies that examined the antidepressant effects of phenolic acids and their potential mechanism of action.

## 2. Phenolic Acids

As discussed above, phenolic acids compose one of the major classes of phenolic compounds. They are present in vegetables, grains, fruits, and other foods and beverages, e.g., teas and spices [36].

**Figure 3:**
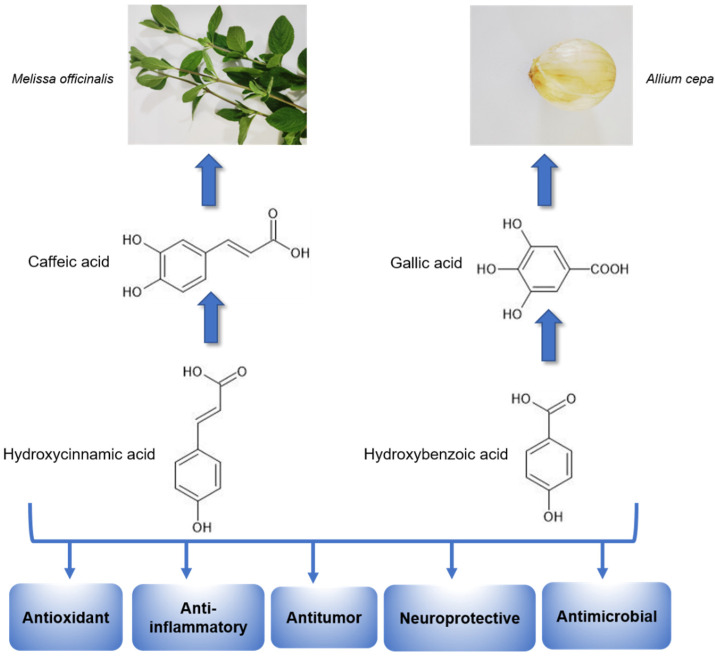
Phenolic acids and their pharmacological properties. This image shows the chemical structures of the two main classes of phenolic acids. They are exemplified by caffeic acid (representative derived from cinnamic acid) and gallic acid (representative derived from hydroxybenzoic acid). The schematic representation shows the sources of these bioactive molecules. Caffeic acid has been isolated from medicinal plants, such as *Melissa officinalis* (lemon balm). Gallic acid is found in fruits and vegetables, such as *Allium cepa* (onions). Phenolic acids (hydrocinnamic and hydroxybenzoic acids) are a group of bioactive molecules that have several pharmacological effects, including antioxidant, antiinflammatory, antitumor, neuroprotective, and potentially antimicrobial, among others. The chemical structures were drawn using ChemSketch freeware, version 14.0 from ACD/Labs.

Based on their chemical structure (Figure 3), phenolic acids may be placed in various classifications. Hydroxycinnamic acids have nine carbon atoms and include several simple phenolic compounds derived from cinnamic acid, including p-coumaric acid, caffeic acid, ferulic acid, chlorogenic acid, and sinapic acid. Hydroxybenzoic acids have seven carbon atoms and consist of compounds derived from benzoic acid, such as gallic acid, salicylic acid, protocatechuic acid, ellagic acid, gentisic acid, and syringic acid [30,37,38] Furthermore, phenolic acid can be in a free form, or it may be associated with different molecules, including simple sugars, organic acids, and plant polymers [37,38].

Numerous in vitro and in vivo studies have shown that phenolic acids may promote pharmacological effects, such as antioxidant [39,40], antitumor [41,42,43], antibacterial [44], antiviral [45], antiinflammatory [46,47], hypoglycaemic [48], anticoagulant [49], antidepressant [50,51], and anxiolytic [52] potential (Figure 3). These results highlight the importance of better understanding the mechanisms by which the biomolecules produce these responses and better use of these molecules as potential new drugs.

### 2.1. Antidepressant Effects of Phenolic Acids

Based on the literature, phenolic acids have potential biotechnological effects because of their pharmacologically bioactive molecules that act on several pathologies. They have neuroprotective effects in conditions such as neuroinflammation, apoptosis, glutamate-induced toxicity, epilepsy, depression, trauma-induced imbalance, brain injury, memory loss, and Parkinson’s disease [27,28]. Laboratories worldwide have been working in this arena to search for possible mechanisms associated with these effects and to characterize their depressive effects using animal models (Table 1). This knowledge could lead to significant advances in drug development, which produce faster and more effective effects in people who develop resistance to antidepressants [14,53]. The focus of this review is to describe some phenolic acids that may play a role in the treatment of depression.

**Figure 4:**
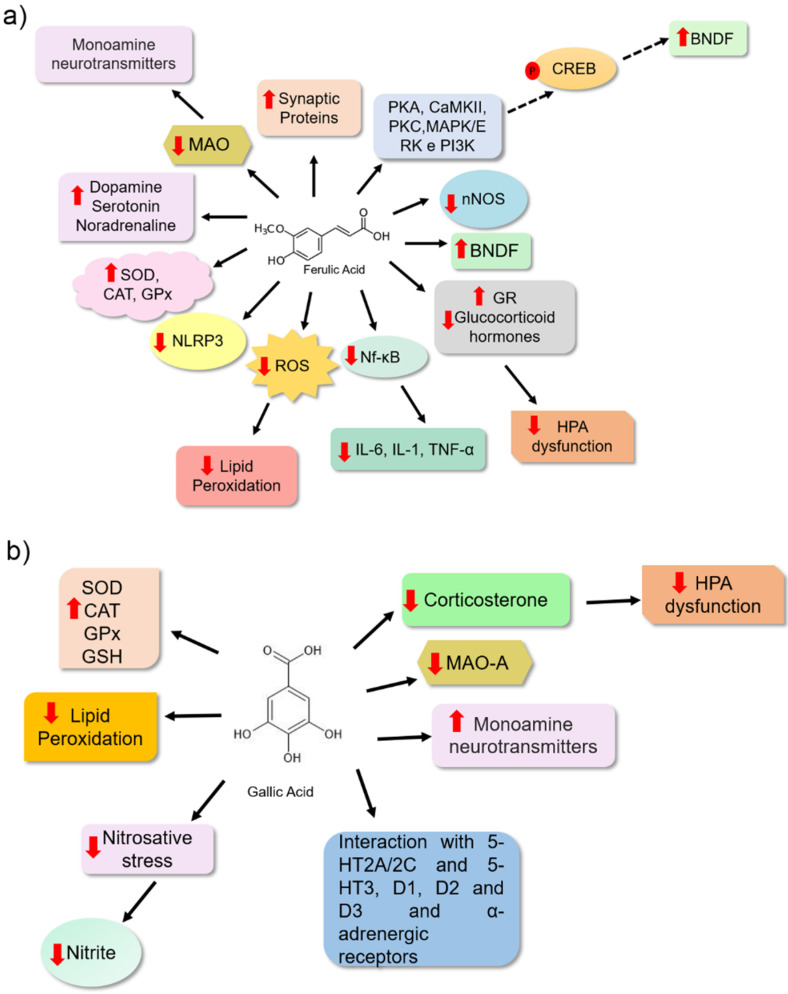
Possible mechanisms of action for ferulic acid (**a**) and gallic acid (**b**) as antidepressants based on preclinical trials. (**a**) Ferulic acid may act by inhibiting MAO-A enzyme activity by increasing levels of endogenous antioxidants, combating lipid peroxidation, inhibiting neuroinflammation, and the hyperactivity of the hypothalamic–pituitary–adrenal (HPA) axis, and increasing levels of brain-derived neurotrophic factor (BDNF) and synaptic plasticity. (**b**) The antidepressant effects of gallic acid are mainly caused by its antioxidant potential because this promotes the activity of antioxidant enzymes, such as SOD, CAT, and GPx, and increases the content of endogenous antioxidants, such as GSH. The compound also inhibits lipid peroxidation and reduces nitrite levels, demonstrating its action against oxidative and nitrosative stress. Additionally, gallic acid may promote the regulation of the hypothalamic–pituitary–adrenal (HPA) axis because it can reduce elevated corticosterone levels. Its effect is also associated with increased serotonin noradrenaline and dopamine levels in the synaptic cleft. These bioactive molecules may interact with alpha-adrenergic, dopaminergic, and serotoninergic receptors to promote their antidepressant effects. Arrows in red (up) indicate increased levels and (down) reduced levels. The solid black arrow indicates stimulation, and the dashed black arrow indicates possible activation (a mechanism not directly evaluated in the study). The chemical structures were drawn using ChemSketch freeware, version 14.0 from ACD/Labs.

Ferulic acid is a bioactive molecule from phenolic acids with a significant antidepressant potential and is one of the most studied for its pharmacological activities. This molecule is present in cereal grains and is an important food source [54]. It has been proposed that ferulic acid (Table 1) may reduce depressive behaviour in animals by inhibiting the monoamine oxidase enzyme (MAO). This leads to the increased availability of monoamine neurotransmitters at the synaptic cleft. Chen et al. [55] observed a reduction in MAO enzyme activity, especially the MAO-A isoform, in the frontal cortex and hippocampus of male ICR (Institute of Cancer Research) mice treated with this compound. They observed that ferulic acid (40 and 80 mg/kg) reduced immobility time in the tail suspension and forced swimming test (20–80 mg/kg). These data suggest that this bioactive molecule may be associated with reduced depressive-like behaviour in animals. These effects have been associated with an increase in 5-HT and NE levels observed in the brains of the animals (Figure 4a).

The brain is constantly subjected to oxidative and nitrosative stress owing to its high demand for oxygen and its rich lipid environment. In the case of a failure in endogenous antioxidant defence, high levels of free radicals may promote lipid peroxidation, and neuronal cells may suffer damage [56,57]. The antidepressant effect of ferulic acid has been linked to its antioxidant potential, as observed in the brains of treated animals. It is a bioactive molecule that may reduce the levels of some oxidative stress markers (malondialdehyde, nitrite, and carbonylated protein) and increase the level of non-protein sulfhydrates (NPSH) (Figure 4a) [58]. Xu et al. [59] also observed a potential antidepressant effect of ferulic acid in animals, related to its antioxidant and antiinflammatory potential. Furthermore, this molecule may prevent neuronal cell apoptosis, and it may have been positively regulated by the level of monoamines in the frontal cortex and hippocampus of the animals that had received 40 and 80 mg/kg. Liu et al. [60] proposed an increase in the prefrontal cortex and hippocampus of mice based on the levels of brain-derived neurotrophic factor (BDNF), which may also be associated with the antidepressant effects of ferulic acid. BDNF plays an important role in neurogenesis and neuronal survival (Table 1). Zeni et al. [61] investigated the pathways involved in the effect of ferulic acid using tail suspension experiments. They identified signalling pathways involved in the pathophysiology of depressive disorders, such as those related to the cellular responses of neuroplasticity, survival, and neurogenesis involving proteins, including protein kinase A (PKA), calmodulin-dependent protein kinase (CaMKII), protein kinase C (PKC), mitogen-activated protein kinase/extracellular signal-regulated kinase (MAPK/ERK), and phosphoinositide 3-kinase (PI3K). When these proteins were blocked, ferulic acid treatment had no effect. These results reinforce the importance of these proteins in the ferulic acid response. The authors suggested that this phenolic acid might also activate the cAMP response element-binding protein (CREB), a transcription factor that upregulates BDNF expression, thereby promoting neurogenesis (Figure 4a) [62].

Several studies have shown an association between neuroinflammation and depression. This correlation is based on studies that showed elevated levels of inflammatory mediators, such as interleukin-6 (IL-6), interleukin-1β (IL-1β), tumour necrosis factor-α (TNF-α), and nuclear factor B (NF-κB), lead to the development of depressive-like behaviour in animals and the elevated levels of these proteins in depressed patients. Additionally, studies have shown that the use of antidepressants leads to reduced levels of proinflammatory cytokines, and the use of antiinflammatory drugs also reduces depressive behaviour in animal models [63,64]. Another molecular mechanism associated with depression is the dysfunction of the hypothalamic–pituitary–adrenal (HPA) axis. It is characterized by elevated glucocorticoid hormones [8,65]. Zheng et al. [47] showed that the antidepressant effect of ferulic acid on Sprague Dawley rats was associated with its antiinflammatory effect, as observed by the reduction in protein concentration and reduction in mRNA expression of proinflammatory cytokines (IL-6, IL-1, and TNF-α). Moreover, it was associated with the inhibition of NF-κB activation, an important transcription factor that modulates proinflammatory cytokine expression and reduces neuronal nitric oxide synthase expression (nNOS). Ferulic acid also reduced adrenocorticotrophic hormone (ACTH) and corticosterone serum levels and increased glucocorticoid receptor (GR) expression, which plays an important role in the feedback regulation of the HPA axis in the hippocampus of animals subjected to prenatal stress. These results suggest an effect of FA on HPA modulation. Liu et al. [66] also proposed that the antiinflammatory activity of ferulic acid might be associated with the reduction in depressive behaviour in animals because of changes in the mRNA expression of L-1β, IL-6, and TNF-α, the reduction in the protein levels of IL-1β and NF-κB, and the inhibition of the NLRP3 inflammasome in the prefrontal cortex of animals. Liu et al. [66] also verified that ferulic acid has an inhibitory effect on microglial activation, an important source of proinflammatory brain mediators (Figure 4a).

Gallic acid is another type of phenolic acid in medicinal plants and fruits that exhibits pharmacological activities, including antiinflammatory, antitumor, and antimicrobial effects. It is also used for metabolic, cardiovascular, gastrointestinal, and neuropsychological disorders [27,67]. The antidepressant effect of gallic acid was evaluated in Sprague Dawley rats for 28 days at 50 and 100 mg/kg (Table 1). This bioactive molecule reduced the immobility time of the animals in the forced swimming assay. The reduction in depressive behaviour has been correlated with antioxidant properties. In brain homogenates from animals treated with gallic acid for 28 days, the inhibition of lipid peroxidation, probably because of the reduction in malondialdehyde (MDA) levels and an increase in the endogenous antioxidant enzymes system, e.g., catalase (CAT) and glutathione peroxidase (GPx), were observed [68]. Corroborating these results, gallic acid administered (25 and 50 mg/kg) to BALB/c mice reduced the depressive-like behaviour of these animals in the forced swimming and tail suspension tests. Furthermore, an increase in endogenous antioxidant defence was observed through the increase in superoxide dismutase enzyme (SOD) and decrease in glutathione enzyme (GSH), and a consequent reduction in lipid peroxidation by thiobarbituric acid reactive substances (TBARS) in mouse brain homogenates (Figure 4b) [69].

Chhillar and Dhingra [70] correlated the neuroprotective effects of gallic acid with its antioxidant potential. Stressed animals treated with gallic acid showed a reduction in the level of corticosterone stress hormone. Furthermore, 10 and 20 mg/kg gallic acid doses reduced MAO-A enzyme activity and nitrite levels in the plasma (Table 1). Can et al. [18] showed that BALB/c mice that received gallic acid (60 mg/kg) exhibited a reduction in immobility time in the tail suspension and a change in their forced swimming pattern. This study proposed that gallic acid may increase monoamine levels at synapses. Furthermore, treatment with alpha-adrenergic, serotoninergic (5-HT2A/2C and 5-HT3), and dopaminergic (D1, D2, and D3) receptor antagonists eliminated the antidepressant-like effect of this bioactive molecule. This showed that one of the antidepressant mechanisms of gallic acid might be associated with these receptors (Figure 4b). The authors also suggested that the effect on monoaminergic neurotransmission may be associated with its potent antioxidant potential.

**Figure 5:**
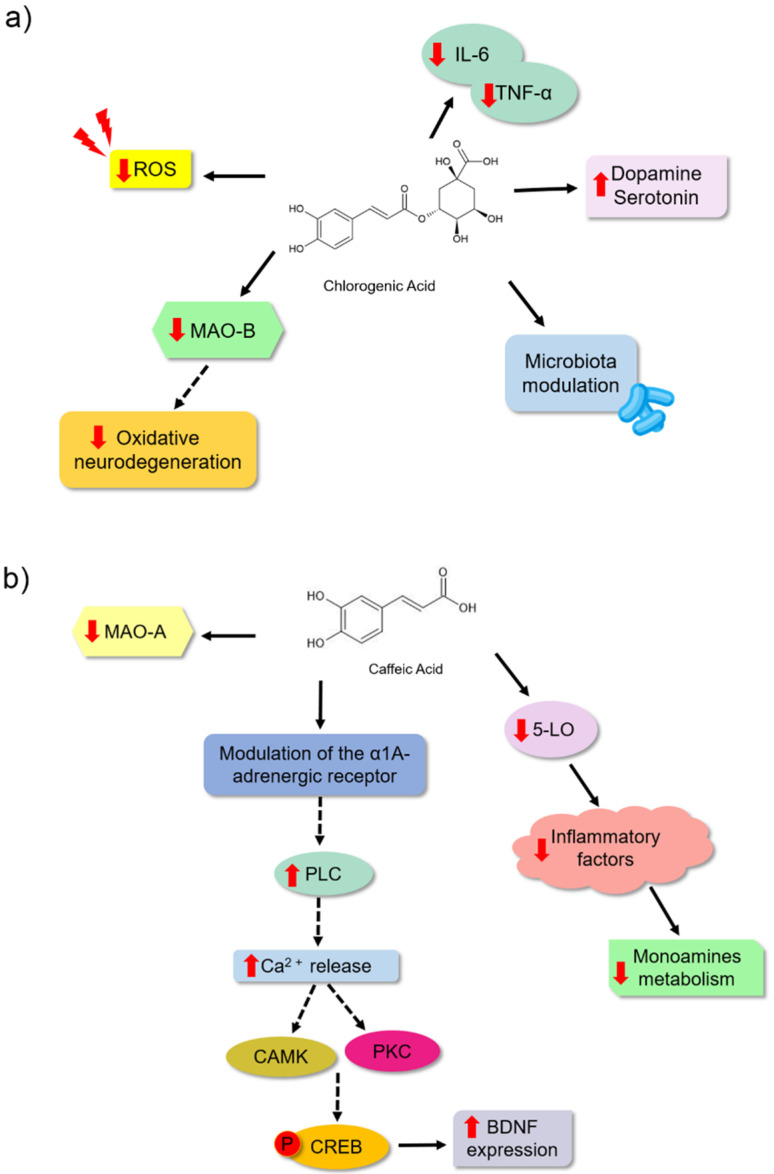
Possible mechanisms of action for chlorogenic acid (**a**) and caffeic acid (**b**) as antidepressants based on preclinical trials. (**a**) Chlorogenic acid may act by increasing levels of monoamine neurotransmitters as an inhibitor of monoamine oxidase isoform B, reducing neuroinflammation, reducing reactive oxygen species production, and improving host gut health by modulating the microbiota. (**b**) Caffeic acid has been shown to act through the modulation of α1 adrenergic receptors. Takeda et al. [78] suggest that the activation of this receptor, which has a stimulator effect on cell signaling by increasing intracellular phospholipase C (PLC), promotes an increase in Ca^2+^ concentration, thereby activating Ca^2^/calmodulin-dependent protein kinase (CAMK) and protein kinase C (PKC). These proteins promote phosphorylation of the cAMP response element-binding protein (CREB). Then, CREB regulates BDNF transcription [78]. Another action mechanism might be its inhibitory effect on the inflammatory lipoxygenase (5-LO) pathway, which may reduce monoamine neurotransmitter levels in the synaptic cleft. Additionally, this bioactive molecule might promote the inhibition of the activity of the MAO-A enzyme. The red arrows (up) indicate increased levels and (down) decreased levels. The solid black arrow indicates stimulation, whereas the dashed black arrow indicates possible activation (mechanism not directly evaluated in the study). The chemical structures were drawn using ChemSketch freeware, version 14.0 from ACD/Labs.

Furthermore, studies have suggested that depression might be a disorder associated with changes in the abundance and diversity of gut microbiota. It is unclear if dysbiosis is a cause or consequence [9,71] of depression. Bidirectional communication in the gut–brain axis has been demonstrated. This communication allows an influence on brain processes since gut bacteria can modulate the immune response, the production of proinflammatory and antiinflammatory cytokines, and tryptophan metabolism, thereby regulating serotonin synthesis, metabolite production, and neurotransmitter levels. Moreover, it has been shown that dysregulation of the HPA axis leads to elevated levels of cortisol, which might modulate bacterial population diversity by promoting an effect on intestinal mucus secretion [71,72,73,74]. Chlorogenic acid is another phenolic acid that has been studied for its effects on depression. It is mostly consumed in coffee because of the high concentrations [75,76]. This bioactive molecule could be a natural antidepressant associated with different mechanisms, among them is the modulation of the gut microbiota (Figure 5a). Song et al. [9] showed that male *Wistar* rats treated with chlorogenic acid (500 mg/kg) exhibited reduced depressive-like behaviour in the sucrose preference assay and a reduced tail suspension and forced swimming experiments. This bioactive molecule could modify the composition of the gut microbial community in these animals. It changed the composition of important bacteria, such as Desulfovibrionaceae, Desulfovibrio, Klebsiella, Burkholderiale, and Bifidobacterium. It inhibited neuroinflammation because it reduced the levels of proinflammatory cytokines (IL-6 and TNF-α) and increased the serotonin and dopamine concentrations in the blood of these animals (Table 1). Lim et al. [77] observed that an extract of a plant with chlorogenic acid could prevent astrocytic hypertrophy in the hippocampus of animals. Furthermore, they observed that when IRC male mice were treated with 30 mg/kg of chlorogenic acid, these animals exhibited a reduction in the time for tail suspension and forced swimming. These authors proposed that this bioactive molecule may be an excellent antidepressant and may act by preventing the reduction in the number of dendritic spines of hippocampal neurons in the spine, which is related to synaptic weakening. Moreover, in vitro assays showed the ability of chlorogenic acid (1 and 10 µM) to specifically inhibit the MAO-B enzyme and reduce the ROS levels in the C8-D1A astrocyte cell lineage (Table 1), thereby protecting against neuronal oxidative stress.

Caffeic acid is a hydroxycinnamic acid present in most vegetables [30]. Takeda et al. [79] observed a significant reduction in the immobility time of animals treated with the highest dose analysed (4 mg/kg) (Table 1). This study showed the ability of this bioactive molecule to inhibit mitochondrial MAO-A activity by 35.5% without inhibiting the B isoform of the enzyme. However, inhibition of the synaptosomal reuptake of the monoamine serotonin, noradrenaline, and dopamine was not observed when compared to the inhibitors used as controls. Considering these results, the authors proposed that the antidepressant properties of this bioactive molecule might be explained by a mechanism other than the inhibition of brain monoamine transporters or MAO. Takeda et al. [80] also evaluated the effect of caffeic acid (4 mg/kg) on depressive behaviour in ICR and ddY mice. They observed that caffeic acid reduced the duration of the immobility of the mice in the forced swim, which the α1A adrenergic receptor may indirectly modulate (Figure 5b). In another study [78], the effect of caffeic acid (4 mg/kg) in male ICR mice was evaluated using a forced swim assay and a reduction in depressive behaviour was observed. These results showed that caffeic acid treatment inhibited the decreased BDNF mRNA expression in the frontal cortex, which might be related to the antidepressant effect of caffeic acid. Figure 5b shows a schematic representation of the two studies by the Takeda group, wherein they proposed that caffeic acid may act by modulating adrenoreceptors. This modulation might activate a molecular cascade that promotes the expression of BDNF. However, these authors stated that more work should be conducted to clearly understand the molecular mechanism by which caffeic acid is involved in preventing a reduction in BDNF expression [81].

In addition to the antioxidant effect of caffeic acid, its antiinflammatory effect may also be related to its antidepressant potential. Huang et al. [82] found that caffeic acid treatment at 10 mg/kg reduced depressive behaviour in Sprague Dawley rats during a forced swimming assay. Inhibition of the enzyme lipoxygenase (5-LO) was observed in this treatment. This enzyme is involved in the development of inflammation, and caffeic acid may act on the metabolism of monoamine neurotransmitters in the cerebral cortex. This reduction is associated with the pathogenesis of depressive disorder. Huang et al. [82] also observed that caffeic acid was able to inhibit the presence of some metabolites, such as 3-methoxy-4-hydroxyphenylglycol (MHPG; metabolite from noradrenaline degradation), 3,4-dihydroxyphenylacetic acid (DOPAC; a metabolite of the dopamine neurotransmitter), 5-HIAA (metabolite from serotonin degradation), and tyrosine and tryptophan (amino acid precursors of noradrenaline and adrenaline and serotonin). Based on these results, the authors proposed that 5-LO inhibition by caffeic acid could affect noradrenaline and serotonin synthesis, affecting the metabolism of other neurotransmitters (Figure 5b).

**Figure 6:**
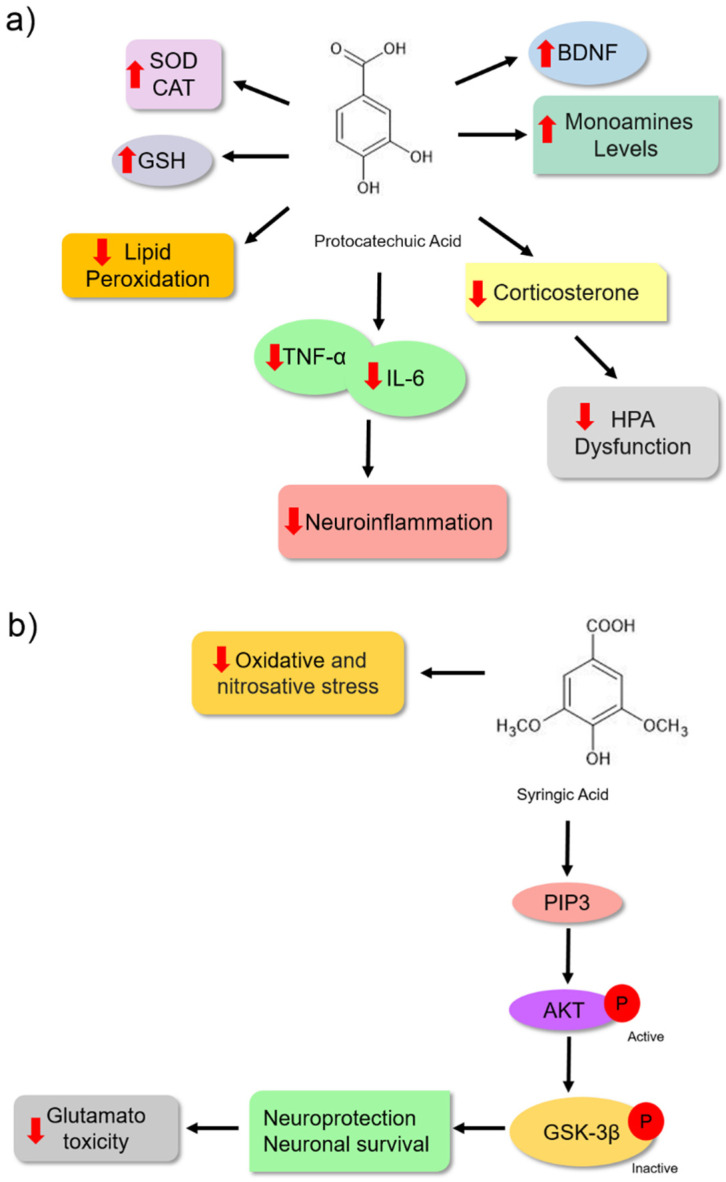
Possible mechanisms of action for protocatechuic acid (**a**) and syringic acid (**b**) as antidepressants based on preclinical trials. (**a**) Protocatechuic acid has been shown to have a depressant effect mainly through antioxidant mechanisms. It increases the activity of the enzymes catalase (CAT), superoxide dismutase (SOD), and the amount of glutathione reductase (GSH). Then, it can prevent lipid peroxidation and inhibit oxidative stress. The antidepressant effect of the bioactive molecule is possible because of its potential to reduce neuroinflammation by inhibiting the deregulation of the hypothalamic–pituitary–adrenal (HPA) axis, increasing brain monoamines, and promoting an increase in BDNF levels. (**b**) Syringic acid promotes antidepressant effect by its antioxidant potential, reducing oxidative stress and nitrosative stress. Another important antidepressant mechanism of this molecule may be associated with glutamatergic excitotoxicity inhibition by PI3K/Akt/GSK-3β neuronal survival pathway activation. Arrows in red (up) indicate increased levels and (down) reduced levels. The solid black arrow indicates stimulation, and the dashed black arrow indicates the possibility of activation (mechanism not directly evaluated in the study). The chemical structures were drawn using ChemSketch freeware, version 14.0 from ACD/Labs.

Protocatechuic acid (PCA) is another phenolic acid whose pharmacological effects are associated with its antioxidant characteristics. These bioactive molecules have antioxidant, antiinflammatory, antibacterial, antidiabetic, anticancer, antiaging, antiviral, antiulcer, hepatoprotective, nephroprotective, and neuroprotective activities [83]. Some data suggest that the antidepressant effect of PCA is also associated with its antioxidant potential. Thakare et al. [84] treated albino Swiss mice with PCA (100 and 200 mg/kg). They observed an antidepressant effect in these animals using the forced swimming assay (Table 1). This effect was associated with a reduction in lipid peroxidation (reduced MDA formation) and increased antioxidant activities of the SOD and CAT enzymes in the hippocampus and cerebral cortex, probably protecting the animals from oxidative damage. Another mechanism observed was the reduction in HPA axis dysfunction, which was verified by a decrease in corticosterone levels in the blood of the animals (Figure 6a).

Additionally, Thakare et al. [85] treated *Wistar* rats with PCA (100 and 200 mg/kg) for 14 days and observed a reduction in lipid peroxidation by the reduced MDA levels in the cortex and hippocampus of the treated animals. The hormone corticosterone was reduced; however, they showed an improvement in CAT and GSH activities, reinforcing the idea of protection against oxidative damage. Moreover, antiinflammatory activity was observed to reduce the levels of proinflammatory cytokines TNF-α and IL-6 (Figure 6a). Pretreatment with PCA also led to increased levels of serotonin, dopamine, noradrenaline, and BDNF. These mechanisms might also be associated with antidepressant effects. Although [85] did not evaluate the MAO enzyme, they proposed an increase in monoamine levels related to the inhibitory effect of enzyme isoforms A and B based on the results of Kim et al. [86]. The antidepressant activity of PCA may be associated with its antioxidant and antiinflammatory activities (Figure 6a).

Glutamate is a neurotransmitter that exerts an excitatory effect on the central nervous system during chemical synapses. However, when present in large concentrations in the synaptic cleft, this agent uncontrollably activates N-methyl-D-aspartate (NMDA) receptors in post-synaptic neurons, causing neuronal damage. Considering the neurotoxic effects of glutamate, dysregulation of glutamatergic neurotransmission has been associated with the development of neurological diseases, psychiatric disorders, and mood disorders, such as depression [87,88,89].

The antidepressant effect of syringic acid on depressive-like behaviour in male Swiss mice was observed through altered forced swimming and tail suspension patterns. The modified pattern was associated with a significant decrease in glutamate-induced toxicity in the hippocampus and cortex of animals (Table 1). Additionally, the study revealed that the antioxidant properties also contributed to its antidepressant effect because treatment with syringic acid led to a reduction in oxidative stress markers, such as serum levels of TBARS, protein carbonyl, and nitrite production in the serum and brain (Figure 6b) [90]. Dalmagro et al. [51] investigated the neuroprotective mechanism of syringic acid on glutamate-induced toxicity in Swiss male mice treated with glutamate at a dose of 1 mg/kg for 7 d. This bioactive molecule might protect hippocampal and cerebrocortical slices against glutamate-induced damage and cell death. Moreover, the neuroprotective effect could be associated, at least in part, with the inhibition of the effects of glutamate on the neuronal cell survival signalling pathway PI3K/Akt/GSK-3β. When activated, the protein phosphatidylinositol 3-kinase (PI3K) phosphorylates Akt, which becomes active, and phosphorylates the GSK-3β enzyme, which becomes inactive. Once inactivated, this enzyme may not promote neuronal cell death (Figure 6b) [91].

**Figure 7:**
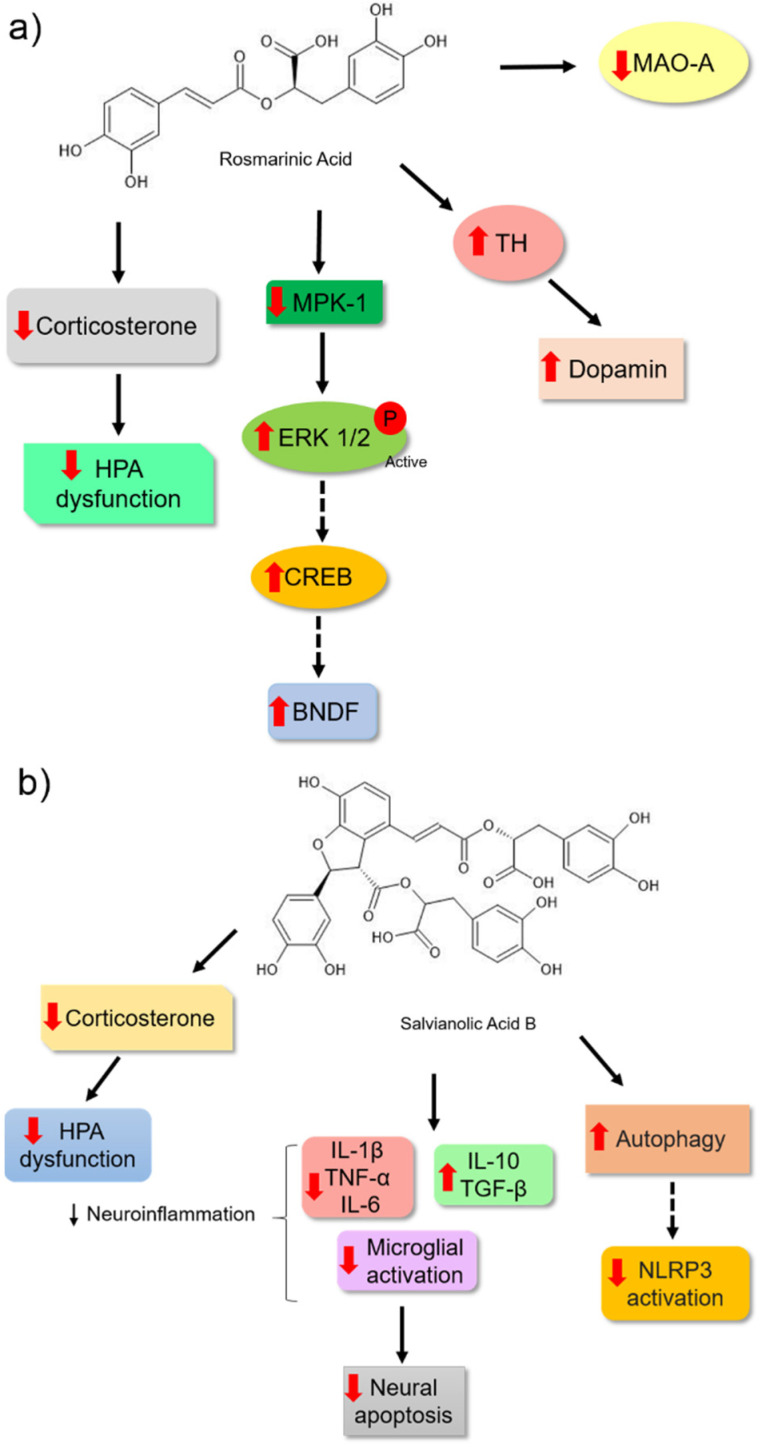
Possible mechanisms of action for rosmarinic acid (**a**) and salvianolic acid B (**b**) as antidepressants based on preclinical trials. (**a**) The rosmarinic acid action mechanism might be associated with inhibiting monoamine oxidase-A enzyme, thereby increasing BDNF expression in the hippocampal region by negative regulation of MKP-1. This may promote stimulation in hippocampal neurogenesis. Furthermore, it may increase tyrosine hydroxylase expression resulting in increased dopamine and decreased corticosterone levels. This may also be associated with the antidepressant effect of this molecule. (**b**) The antidepressant effect of salvianolic acid B may be associated with its antiinflammatory effect because this molecule can suppress the expression of proinflammatory cytokines and increase the expression of antiinflammatory cytokines. This response protects against apoptosis and hippocampal atrophy promoted by neuroinflammation. Its antidepressant effect may be associated with the inhibition of microglia and NLRP3 inflammasome activation and autophagy. Additionally, it might modulate corticosterone levels with the normalization of HPA axis hyperactivity, which may be associated with its antidepressant action. Arrows in red (up) indicate increased levels and (down) reduced levels. The solid black arrow indicates stimulation, and the dashed black arrow indicates the possibility of activation (mechanism not directly evaluated in the study). The chemical structures were drawn using ChemSketch freeware, version 14.0 from ACD/Labs.

Rosmarinic acid is another bioactive molecule from phenolic acids that has been identified in several aromatic herbs used in traditional medicine and cooking, such as rosemary, sage, mint, basil, and oregano [92]. Takeda et al. [79] observed that rosmarinic acid showed an antidepressant effect in ICR mice because it changed the pattern in the forced swim assay (Table 1). This molecule was able to weakly inhibit the activity of MAO-A. However, it did not affect the synaptosomal uptake of monoamines in the brains of the animals. The authors hypothesised that this bioactive molecule might be acting by other mechanisms. Moreover, rosmarinic acid was able to positively modulate hippocampal neurogenesis by increasing the proliferation of hippocampal dentate gyrus cells, an effect that may have been associated with reduced immobility time in the forced swim test of male ddY mice in a dose-dependent manner [93]. In support of these results, Ref. [94] observed that chronic treatment of male Sprague Dawley rats for 14 d with the highest dose of rosmarinic acid (10 mg/kg) reduced their depressive behaviour. This result was correlated with the increase in the expression of the active extracellular signal-regulated kinase (pERK1/2) and BDNF levels in astrocytes and the hippocampal tissue of the treated rats. Therefore, the effects of rosmarinic acid may be blocked by treatment with a selective inhibitor of ERK1 and ERK2 (U0126), which suggests that rosmarinic acid may induce BDNF expression through the ERK signalling pathway and plays an important role as a potential antidepressant (Figure 7a).

Additionally, Kondo et al. [95] provided important insights into the possible molecular mechanisms by which rosmarinic acid may act as an efficient antidepressant. They observed a reduction in the serum levels of corticosterone. These data suggested that this molecule may influence the normalisation of HPA hyperactivity. The authors also observed a positive regulation of BDNF levels in animals treated with rosmarinic acid. The effect was associated with the reduced expression of MAPK phosphatase-1 (MPK-1). High levels of MKP-1 in the brain may inhibit ERK1/2 kinase activity through its dephosphorylation. When this protein is inactive, it may not phosphorylate other proteins, such as the transcription factor CREB, which in turn may not activate target genes in a cascading manner, such as BDNF [96,97]. Considering the importance of monoamine deficiency in the pathology of depressive disorder, the authors correlated the antidepressant potential of rosmarinic acid with an increase in dopamine levels in the brain. This may be related to the increase in the tyrosine hydroxylase (TH) enzyme, which plays an important role in monoamine synthesis (Figure 7a).

Salvianolic acid B is a natural polyphenol in *Salvia miltiorrhiza* and is widely used in traditional Chinese medicine. This phenolic acid has different pharmacological and antidepressant effects (Table 1) [98,99]. The antidepressant action of 20 mg/kg salvianolic acid B in C57BL/6 mice was correlated with the reduction in corticosterone levels in the plasma, mRNA expression, and proinflammatory cytokine levels, and an increase in antiinflammatory cytokine mRNA expression in the hippocampus and cortex of the animals (Figure 7b). Moreover, salvianolic acid B could also regulate microglial activation and block apoptosis in the hippocampus and cerebral cortex, thereby avoiding the reduction in the volume and cell density of the hippocampus after 3 weeks of treatment. This result reinforces the idea that this effect is associated with inhibiting the neuroinflammatory process, and it could reactivate the HPA axis and prevent neuronal loss (Figure 7b) [98].

Additionally, Jiang et al. [100] measured the antidepressant effect of salvianolic acid B (20 mg/kg) in a lipopolysaccharide (LPS)-induced neuroinflammation Sprague Dawley mice model. They observed that phenolic acid could reduce the behavioural patterns and neuroinflammation induced by LPS in the brains of animals. The authors observed a reduction in the expression of proinflammatory cytokines and the activation of the NLRP3 inflammasome by a reduction in the expression of NLRP3, the adaptor protein ASC, and caspase-1 P20 (Figure 7b). Furthermore, this bioactive molecule restored autophagy, which was reduced in the hippocampus, and increased autophagy was verified by the expression of biomarkers (LC3-II/I and Beclin-1). These markers are reduced during neuroinflammation of the hippocampus. During the development of some neurological disorders, such as depression, autophagy is modified [101,102], which could indicate parts of the antidepressant effects of this compound. Additionally, the study showed a reduction in microglial activation, supporting the results of [98].

**Figure 8:**
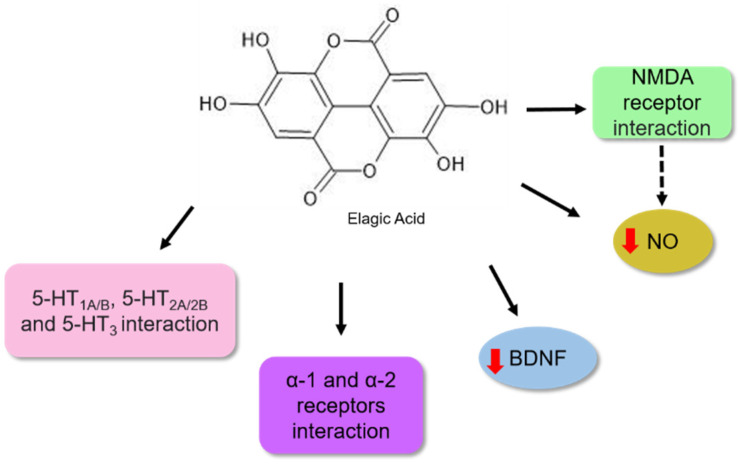
Possible mechanisms of action for ellagic acid as antidepressant based on preclinical trials. Studies have shown an association between serotoninergic, 5-HT1A/B, 5-HT2A/2B, and 5-HT3 receptors, and α-1 and α-2 adrenergic receptors in the antidepressant action of ellagic acid. Ellagic acid increased BDNF levels in the hippocampus. Its effect on the glutaminergic NMDA receptor was verified by modulating the expression of NR2A and NR2B subunits. This bioactive molecule may reduce the NO levels. This molecule may inhibit the NMDA-NO pathway and reduce nitric oxide levels, playing a part in the ellagic acid antidepressant effect. The red arrows (up) indicate increased levels and (down) reduced levels. The solid black arrow indicates stimulation, and the dashed black arrow indicates the possibility of activation (mechanism not directly evaluated in the study). The chemical structures were drawn using ChemSketch freeware, version 14.0 from ACD/Labs.

Ellagic acid is a common phenolic acid in fruits, such as raspberries, strawberries, and blackberries [103]. Its pharmacological activities are associated with antiobesity, antiviral, antioxidant, and antitumour effects [104]. Girish et al. [105] described its potential as an antidepressant. According to these authors, the bioactive molecule reduced immobility time in forced swimming and tail suspension assays in mice. It has been proposed that this activity might be associated with the interaction of this bioactive molecule with noradrenergic systems, involving α-1 and α-2 adrenergic receptors and 5-HT1A/B, 5-HT2A/2B, 5-HT3, the serotoninergic receptors. In contrast, no participation in the opioid system was observed (Figure 8). 

Bedel et al. [106] observed that when BALB/c mice were treated with ellagic acid at concentrations of 1, 2.5, and 5 mg/kg for 14 days, a reduction in depressive behaviour was observed in the tail suspension and forced swimming analysis (Table 1). The authors proposed that the antidepressant potential might be associated with an increase in BDNF levels in the hippocampus, as shown in Figure 8. 

Lorigooini et al. [107] observed an antidepressant effect in a dose-dependent manner when treated with ellagic acid. The authors proposed that the possible mechanism involved the blockade of NMDA receptors because it could modulate the expression of NR2A and NR2B subunits of the NMDA receptor in the hippocampal cells of the animals. Moreover, a reduction in the nitric oxide (NO) level was also observed in the hippocampus. NO is a neuromodulatory molecule that can diffuse between membranes and modulate neurotransmitter systems in neuronal cells. NO synthesis occurs because of the activation of the NO synthase enzyme, resulting in an influx of calcium triggered by NMDA receptor activation [108]. Based on their results, the authors proposed that the antidepressant effect of ellagic acid observed in behavioural assays might be associated, in part, with the suppression of the NMDA-NO pathway (Figure 8) [107].

The results regarding depressive behaviour in animals described in the various studies demonstrated the effects of isolated phenolic acids. The antidepressant effects of the different pathways of extracts obtained from herbal medicinal plants with more highly concentrated bioactive molecules remain unclear. However, some studies associate part of this effect to the presence of these compounds.

### 2.2. Antidepressant Potential of Medicinal Plants Rich in Phenolic Acids 

Plants have been used in folk medicine for a long time. Consequently, research on bioactive molecules to identify and characterise old or new bioactive molecules is an important field as they may be used as alternatives for depression treatment, thus having an important biotechnological potential [109]. Some extracts have been evaluated in preclinical studies, such as *Melissa officinalis*, which may be considered to have an effect in depression as it changed the swimming pattern. This effect was according to the extract concentration used, for example, it worked as acute (300 mg/kg) and as subacute (30, 100 and 300 mg/kg) [110]. The authors raised the hypothesis that the bioactive molecules could be acting on the serotoninergic system. It positively regulated serotonin retention and had an MAO-A inhibitory effect, as reported in previous studies. In addition, HPLC analysis showed the high presence of rosmarinic acid in the leaf extract, associated with its potential for depressant treatment.

The other extract evaluated was the aqueous extract of the leaves and roots of *Taraxacum officinale.* Li et al. [111] observed a reduction in depressive behaviour in animals. This reduction was similar to the positive control, which was fluoxetine used in both acute (1 day) and chronic (14 days) treatments. In the highest dose analysed (200 mg/kg) they observed a reduction in serum corticosterone and corticotrophin levels and proposed that the extract might act by regulating the neuroendocrine system.

Furthermore, Ref. [112] observed antidepressant activity with a hydroethanolic extract of the leaves of *Taraxacum officinale*. They proposed that this effect may be associated with the reduction in corticosterone levels and the increase in dopamine, noradrenaline, and adrenaline levels in the brain, and an increase in the expression of the BDNF marker and decrease in Mkp-1 expression. Phytochemical analysis of the extract by LC/MS identified synaptic acid, gallic acid, isoetin, hesperidin, naringenin, and kaempferol as its main components. 

Di Lorenzo et al. [113] used *Aristotelia chilensis* extracts at 25, 50, and 100 mg/kg. They observed an antidepressant effect associated with antioxidant activity because of a reduction in TBARS levels, and an increase in CAT and SOD enzyme activity, and the increase in GSH level was observed in a dose-dependent manner. Analysis by RP-HPLC-PDA-ESI-MS allowed the identification of 32 compounds, including nine anthocyanins, six organic acids and phenolic acids (such as protocatechuic acid and gallic acid), three derivatives of ellagic acid, and 14 flavonoids (quercetin and rutin). The results revealed a correlation between oxidative stress biomarkers and depressive symptoms, suggesting that phenolic compounds reduce depressive behaviour through antioxidant activity.

Another extract from fruits analysed was from *Hypericum androsaemum* L. (15 and 30 mg/kg), and its antioxidant potential and antidepressant effects were verified in male BALB/c mice. The extract composition was determined by HPLC-DAD and showed the presence of catechins (catechin and epicatechin), phenolic acids (chlorogenic acid and gallic acid), and flavonoids (rutin and quercetin). Nabavi et al. [114] observed that the animals treated with this extract showed a reduction in lipid peroxidation (TBARS) and increased CAT and SOD enzyme activity and GSH content. These results showed antioxidant protection. Moreover, Ref. [115] reported a relationship between the antioxidant effect of compounds present in the ethyl acetate leaf extract of *Eugenia catharinensis.* When rats were treated with different concentrations (50, 125, 200, or 250 mg/kg), a reduction in depressive behaviour was observed using different assays, and an increase in antioxidant activity, including a reduction in lipid peroxidation, an increase in SOD and CAT activity in the cerebral cortex, and an increase in SOD and GSH-Px activity were observed in the hippocampus. The HPLC-ESI-MS/MS analysis showed the presence of 15 bioactive molecules, mostly phenolic acids, such as gallic acid, protocatechuic acid, syringic acid, 4-hydroxymethylbenzoic acid, chlorogenic acid, salicylic acid, caffeic acid, vanillic acid, p-coumaric acid, isoquercetin, rutin, ferulic acid, aromadendrin, galangin, and apigenin. Baraúna et al. [115] proposed that this antioxidant potential in the hippocampus and cerebral cortex areas might be associated with the presence of these bioactive molecules, which are responsible for the observed reduction in depressive behaviour of the animals.

The extract from *Micromeria myrtifolia* was evaluated for its antidepressant effects, and it was able to reduce the depressive behaviour of animals. The fractionation of a methanolic extract (100 mg/kg), which had the greatest effect, had two fractions (B and C) that reduced the depressive behaviour of the animals, and its analysis led to the isolation of rosmarinic acid, myricetin, apigenin, and naringenin, which were tested for antidepressant activity by forced swimming and tail suspension tests. The experiments highlighted rosmarinic acid as the most promising compound because it showed activity in both behavioural assays and revealed the ability to inhibit MAO-A and MAO-B enzymes in both the extract and the isolated compound, an effect that would have antidepressant potential [109]. Rahmati et al. [116] also attributed part of the dose-dependent antidepressant effect of the hydroethanolic extract of the aerial parts of *Lavandula officinalis* (200 and 400 mg/kg) in male NMRI rats in the forced swimming assay to the presence of polyphenols, such as rosmarinic acid and caffeic acid.

Daodee et al. [117] evaluated the ethanolic extract of *Dipterocarpus alatus* leaves (100 and 500 mg/kg) by sucrose preference, forced swimming, and tail suspension assays in male ICR mice. The study showed that the extract reduced the depressive behaviour of the animals in both assays, with an effect similar to that of the imidaprine control. HPLC analysis detected the presence of flavonoids and phenolic acids in the leaves of this species, and it was possible to identify the compounds luteolin-7-O-glucoside, kaempferol-3-glucoside, rutin, gallic acid, ferulic acid, and caffeic acid. According to the authors, the reduction in serum corticosterone levels indicated that the antidepressant effect of the extract was related to the suppression of HPA axis hyperactivity. The extract reduced the SGK1 mRNA expression, a molecule that acts by mediating the effects of glucocorticoids on brain neurogenesis [118] and inhibits a stress-induced decrease in CREB and BDNF mRNA expression, thereby providing evidence that enhancement of neurogenesis could be an effect that contributes to the antidepressant properties of the extract. The reduction in MAO-A activity could also be a possible mechanism of action of the extract.

**Figure 9:**
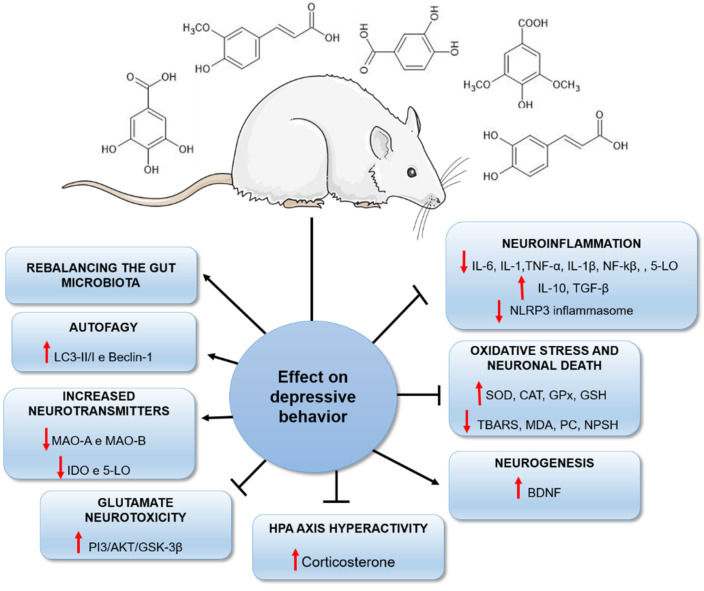
Overall summary, with the main possible molecular mechanisms by which phenolic acids may reduce depressive-like behaviour in rodents. T-shaped arrows stand for inhibition and normal arrows for stimulation. Figure was created using image from Servier Medical Art (https://smart.servier.com) accessed on 16 August 2022, licensed under a Creative Commons Attribution 3.0. The chemical structures were drawn using ChemSketch freeware, version 14.0 from ACD/Labs.

In conclusion, plants are a promising source of biomolecules with important neuroprotective effects [19,25]. Our review reported the potential of a class of natural compounds, the phenolic acids, capable of reducing depressive behaviours by different biological mechanisms (Figure 9). Although the antioxidant and antiinflammatory properties of these agents are primarily responsible for their antidepressant effects, it is important to highlight that other mechanisms may underlie the reduction in depressive behaviour in rodents (Figure 9). This fact should stimulate the development of further studies investigating plants and their isolated constituents, such as phenolic acids, as efficient therapeutic strategies in the future.

## 3. Nutraceutical Perspectives

The individual’s nutritional status has been associated with different neurological disorders, such as depression [119]. Its importance is related to several studies that have shown a relationship between dietary patterns and depression, where a diet poor in certain nutrients may correlate with a higher incidence of the disease [120,121,122,123].

The dietary pattern of patients with depressive disorder is often composed of a low intake of protein and high-quality fats, excessive consumption of proinflammatory foods such as sugars, refined flours, low-quality fats, sugary drinks, and sweets [121,124], which have been shown to correlate with the occurrence of depressive symptoms [125].

Mechlinska et al. [126] observed that patients with depression resistant to treatment with antidepressants also presented a diet based on a low consumption of foods rich in antiinflammatory compounds such as vegetables and a lower frequency in the consumption of olives, grains, avocado, and dairy products than healthy volunteers, leading to the accumulation of adipocytes, affecting brain physiology, which can lead to the occurrence of depression.

Adding nutritional components to the diet is an important strategy to promote mental health and combat depressive symptoms [124]. Adherence to a healthy eating pattern containing a high consumption of fruits, vegetables, and fish and a low consumption of processed meats and red meat has been associated with reduced depressed mood [127]. Thus, adding a diet rich in fruits, nuts, olive oil, fish, legumes, and whole grains has also shown high potential in the prevention and treatment of depression since they present a relevant amount of nutrients with neuroprotective effects, such as omega-3 fatty acids, vitamins B12 and magnesium, zinc and tryptophan, and calcium [124,128]. The implementation of a diet rich in antioxidant and antiinflammatory compounds, such as the Mediterranean diet supplemented with fish oil, was able to improve depressive symptoms significantly, and the mental health and quality of life in patients with depression over three months, and statistically significant correlations were found between improved diet and reduced depression. These dietary improvements were sustained over six months. The study also showed that reducing an inflammatory diet based on eating unhealthy snacks was associated with improved mental health in patients [129].

The evidence that a combination of healthy dietary practices as well as specific nutritional interventions can reduce the risk of developing depression and combat symptoms in depressed patients [119,130] reinforces the potential of nutraceuticals as a non-pharmacological alternative that could help in the treatment of depression, coupled with available pharmacological strategies. Implementing a diet of foods from plants such as fruits and vegetables, as well as teas and coffee, may be necessary as a strategy against depressive disorder since they present bioactive compounds such as polyphenols and phenolic acids, agents with antidepressant effects, already demonstrated in the literature [131].

The several experimental studies employing animal models to evaluate phenolic acids as antidepressant agents, associated with the neuroprotective potential and reduced incidence of the disorder in people whose diet is composed of foods rich in phenolic acids, indicate that the nutraceutical application of these antioxidant and antiinflammatory compounds is beneficial for the treatment of the depressive disorder. However, the importance of future nutritional randomized controlled clinical trials considering important conflicting factors such as study population, double-blind studies, study time, baseline intake, and the status of a nutrient [132,133] that make it possible to determine the effect, dosage, and efficacy of specific phenolic acids on depressive patients, so that they can be used as an ancillary intervention to be explored in the future, is emphasized.

Methods for accurately measuring people’s dietary intake remain problematic. In addition to listing which standard diet to follow as the healthiest, another methodological issue that may obscure and hinder the assessment of the diet–mental health relationship concerns the covariance between health behaviours such as diet, physical activity, and smoking. It is also pointed out that supplements derived from single nutrients would be flawed as a prevention or treatment strategy since nutrients are never consumed in isolation in the diet [134]. Therefore, greater efficacy could be achieved using simultaneous and synergistic combinations of nutraceuticals.

**Table 1:** Effect of phenolic acids on animal models of depression and their mechanisms of action.

Phenolic Acid	Behavioral Analysis	Animal	Dose (per kg)	Time from Treatment	Effects	Reference
Ferulic Acid	Sucrose preference and forced swim tests	Male Sprague Dawley Rats	12.5, 25, and 50 mg	28 days	It reduced the concentration of proinflammatory cytokines IL-6, IL-1β, and TNF-α in the hippocampus, reduced expression of neuronal nitric oxide synthase (nNOS), increased IL-10, reduced ACTH, corticosterone in the hippocampus and increased GR expression.	[47]
Ferulic Acid	Tail suspension test	Male Swiss mice	1 mg	7 days	It reduced markers of oxidative stress (MDA, nitrite, and PC) in the brains of the animals and increased NPSH levels.	[58]
Ferulic Acid	Sucrose preference and forced swim tests	ICR male mice	20 and 40 mg	28 days	It increased the concentration of BDNF, and of the synaptic proteins PSD95, inapsin I in the prefrontal cortex and hippocampus	[60]
Ferulic Acid	Tail suspension and sucrose preference tests	ICR male mice	20, 40, and 80 mg	28 days	It reduced mRNA expression of IL-1β, IL-6, and TNF-α and reduced mRNA expression and protein levels of CD11b, protein levels of NF-κB and IL-1β inhibited the NLRP3 inflammasome in the prefrontal cortex.	[66]
Ferulic Acid	Thermal hyperalgesia, mechanical allodynia, tail suspension, and forced swimming	ICR male mice	5, 10, 20, 40, and 80 mg	30 min before the test	It increased noradrenaline, 5-HT and dopamine in the hippocampus and frontal cortex; reduced lipid peroxidation levels, nitrite, IL-1β, TNF-α in the frontal cortex and hippocampus; increased SOD activity, GSH levels and reduced levels of the neuromodulator substance P, NF-κβ p65, and caspase-3.	[59]
Ferulic Acid	Forced swim test	Male Sprague Dawley Rats	25 and 50 mg	24, 5 and 1 h before the test	It inhibited monoamine reuptake, reduced CRH, ACTH concentrations and increased 5-HT in plasma, prefrontal cortex, and hippocampus of rats.	[135]
Ferulic Acid	Forced swim and tail suspension tests	Male ICR mice	10, 20, 40, and 80 mg	30 min before the test	It increased serotonin and noradrenaline levels in the hippocampus, frontal cortex, and hypothalamus, and inhibited monoamine oxidase-A (MAO-A) activity in the frontal cortex and hippocampus.	[55]
Ferulic Acid	Forced swim and tail suspension tests	Male Swiss mice	0.001, 0.01, 0.1, 1, and 10 mg	60 min before the test	Interacted with the serotonergic system.	[14]
Ferulic Acid	Tail suspension and forced swim tests	Male Swiss mice	0.01, 0.1, 1, and 10 mg	21 days	It increased SOD, CAT, and GSH-Px activities in the cerebral cortex, decreased TBARS levels in animals subjected to stress.	[136]
Ferulic Acid	Tail suspension test	Male Swiss mice	0.01 mg	30 min before the test	It activated the PKA, CaMKII and PKC, MAPK/ERK, and PI3K signaling pathways.	[61]
Gallic acid	Forced swim test	Male Sprague Dawley Rats	50 and 100 mg	28 days	It reduced MDA levels and increased CAT and GPx activity in the brain homogenates of the animals.	[68]
Gallic acid	Tail suspension and forced swim tests	BALB/c mice	25 and 50 mg	7 days	It reduced TBARS levels and increased SOD activity and GSH levels.	[69]
Gallic acid	Tail suspension and modified forced swim tests	Male BALB/c mice	30 and 60 mg	24, 5 and 1 h before the tests	It increased the levels of serotonin and catecholamines in the synaptic clefts of the central nervous system. It also had its effect related to α-adrenergic, 5-HT2A/2C, and 5-HT3 serotoninergic and D1, D2, and D3 dopaminergic receptors.	[18]
Gallic acid	Forced swim and sucrose preference tests	Male Swiss mice	5, 10, 20 mg	21 days	It reduced MAO-A activity, reduced nitrite and malondialdehyde levels in plasma. In addition, it reduced the corticosterone content in the plasma of the mice. It increased the levels of reduced glutathione and catalase activity.	[70]
Chlorogenic acid	Tail suspension and forced swim tests	ICR male mice	10 and 30 mg	7 days	Inhibited the reduction in the number of neuronal dendritic spines, inhibited the enzyme MAO-B and ROS production in hippocampal astrocyte cultures of the animals.	[77]
Chlorogenic acid	Sucrose preference, forced swim and tail suspension tests	Wistar male	500 mg	14 days	Significantly reduced serum levels of the proinflammatory cytokines IL-6 and TNF-α; increased serum concentrations of the neurotransmitters serotonin and dopamine. Modified the structure of the intestinal microbial community of the animals.	[9]
Caffeic acid	Forced swim test	ICR male mice	4 mg	30 min before the test	Attenuated the reduction in BDNF mRNA expression levels in the frontal cortex and TrkB in the mouse amygdala.	[78]
Caffeic acid	Stress tests with conditioned fear and forced swim Forced swim test	ICR male mice and ddY mice	4 mg	30 min before the test	It modulated the α1A adrenergic receptor.	[80]
Caffeic acid	Forced swim test	ICR male mice	1–4 mg	30 min before the test	It slightly reduced the activity of MAO-A.	[79]
Caffeic acid	Forced swim test	Male Sprague Dawley Rats	10 and 30 mg	21 days	It modulated NE and 5-HT synthesis and affected the metabolism of other neurotransmitters through inhibition of the inflammatory 5-Lipoxygenase (5-LO) pathway.	[82]
Protocatechuic Acid	Forced swim test	Swiss albino mice	100 and 200 mg	8 hours and 40 min	Reduced serum corticosterone levels, MDA formation in hippocampus and cerebral cortex; restored SOD and CAT activities in hippocampus and cerebral cortex.	[84]
Protocatechuic Acid	Forced swim test	Wistar rats of both sexes	100 and 200 mg	14 days	It increased the levels of 5-HT, DA, and NE, prevented the reduction in BDNF, prevented the elevation of TNF-α and IL-6 levels, reduced MDA levels and increased CAT activity and GSH content in the hippocampus and cerebral cortex; it reduced the serum corticosterone level in the animals.	[85]
Syringic acid	Forced swim and tail suspension tests	Male Swiss mice	0.1, 1, 10 and 100 mg	Acute (1 time) 60 before the test Subchronic (7 days)	Reduced TBARS levels in serum. Neutralized nitrite production in the serum and brain, reduced protein carbonyl production, and reduced glutamate-induced toxicity in the hippocampus and cortex of the animals.	[90]
Syringic acid	Tail suspension test	Male Swiss mice	1 mg	7 days	It protected hippocampal and cerebrocortical slices against glutamate-induced damage, possibly through the PI3K/Akt/GSK-3β pathway.	[51]
Rosmarinic Acid	Forced swim test	ICR male mice	1–4 mg	30 min before the test	Slightly inhibited the activi- ty of monoamine oxidase-A	[79]
Rosmarinic Acid	Forced swim test andMorris water maze test	Male Sprague Dawley Rats	5 and 10 mg	14 days	It increased hippocampal expression of pERK1/2 and BDNF levels.	[94]
Rosmarinic Acid	Forced swim test	Male ddY mice	1, 2, and 4 mg	7 and 14 days	Positively modulated hippocampal neurogenesis.	[93]
Rosmarinic Acid	Tail suspension test	ICR male mice	5 and 10 mg	7 days	It reduced serum corticosterone levels, increased dopamine, reduced Mpk-1 mRNA expression and increased BDNF mRNA expression, increased tyrosine hydroxylase and pyruvate carboxylase expression.	[95]
Salvianolic acid B	Forced swim and sucrose preference tests	Male Sprague Dawley Rats	20 mg	14 days	It alleviated the increased expression of proinflammatory cytokines, IL-1β and IL-6, reduced the expression of Iba-1, restored the expression of autophagic biomarkers, including LC3-II/I and Beclin-1, in the rat hippocampus and reduced the expression of NLRP3, ASC, caspase-1 P20, components of the NLRP3 inflammasome.	[100]
Salvianolic acid B	Sucrose preference, forced swim and tail suspension tests	Male C57BL/6 mice	20 mg	21 days	It reduced the mRNA expression and protein levels of IL-1β and TNF-α and increased the expression of IL-10 and TGF-β in the hippocampus and cortex of mice. Reduced plasma levels of corticosterone. Prevented apoptosis in the hippocampus and cortex of mice and reduced microglia activation in these brain regions.	[98]
Ellagic Acid	Forced swim test and splash test	NMRI male mice	6.25, 12.5, 25, 50, and 100 mg	60 min before the test	It significantly reduced the level of nitric oxide (NO) in the hippocampus, modulated the expression of NR2A and NR2B subunits of the NMDA-R receptor.	[107]
Ellagic Acid	Forced swim and tail suspension tests	Male BALB/c mice	1, 2.5, and 5 mg	14 days	Increased the levels of BDNF protein in the hippocampus of the animals.	[106]
Ellagic Acid	Forced swim and tail suspension tests	Albino mice	25, 50, and 100 mg	Acute—30 before the tests Chronic—14 days	It modulated the monoaminergic and noradrenergic systems.	[105]

## Data Availability

Not applicable.
